# The value of L3 skeletal muscle index in evaluating preoperative nutritional risk and long-term prognosis in colorectal cancer patients

**DOI:** 10.1038/s41598-020-65091-0

**Published:** 2020-05-18

**Authors:** Shengqiang Wang, Hailun Xie, Yizhen Gong, Jiaan Kuang, Ling Yan, Guotian Ruan, Feng Gao, Jialiang Gan

**Affiliations:** Department of Colorectal Anal Surgery, The First Affiliated Hospital of Guangxi Medical University, Nanning, Guangxi China

**Keywords:** Gastrointestinal cancer, Colorectal cancer, Surgical oncology

## Abstract

L3 skeletal muscle index (L3SMI) was reportedly related to postoperative outcomes. We aimed to investigate the value of L3SMI in evaluating preoperative nutritional risk and long-term prognosis in colorectal cancer (CRC) patients. We retrospectively enrolled 400 CRC patients who underwent surgery from January 2012 to December 2014. The L3SMI was calculated by preoperative computed tomography (CT) and classified into two groups by gender quartile method. We found that the CT diagnostic criteria of sarcopenia in South China population was: male ≤38.89cm^2^/m^2^, female ≤33.28cm^2^/m^2^. Multivariate logistic regression analysis showed that low L3SMI was an independent risk factor for preoperative nutritional risk (p < 0.001). Kaplan-Meier survival curves showed that low status group had significantly lower disease-free survival (p = 0.004) and overall survival (p = 0.001), especially in TNM II stage. Multivariate Cox regression analysis revealed preoperative low L3SMI adversely affected disease-free survival (p < 0.001, HR 1.894 (95% CI: 1.330–2.698)), and overall survival (p < 0.001, HR 2.030 (95% CI: 1.420–2.902)). In conclusion, L3SMI is a useful supplement for screening preoperative nutritional risk and diagnosing sarcopenia, and a potential clinical indicator that can be used to predict the prognosis of CRC patients, especially TNM stage II patients.

## Introduction

Colorectal cancer (CRC) has become the third most common malignant tumor worldwide. According to the 2018 Global Cancer Epidemiological Statistics (GLOBOCAN) database, there are approximately 1.09 million new cases of CRC (morbidity rate of 6.1%), and approximately 551,000 people (mortality rate 9.2%) die from CRC each year, making CRC the second deadliest cancer in the world^1^. CRC has become the fifth most usual malignant cancer and the fourth deadliest malignant cancer in China, with the incidence gradually increasing^2^. At present, surgical resection is still the mainstay curative treatment for CRC, but the 5-year survival rate for large numbers of CRC patients after R0 resection is still unsatisfactory^3,4^. Therefore, prognostic factors for CRC patients are critical in guiding treatment options and follow-up strategies.

In recent years, several studies have shown that sarcopenia and malnutrition seriously influence the quality of life and prognosis of cancer patients^5,6^. Sarcopenia is characterized by a decline in muscle mass and function associated with aging, lack of activity and chronic diseases including various malignancies. Skeletal muscle reduction is a key feature of sarcopenia, which is also associated with poor nutrition and survival prognosis in cancer patients^7^. Malnutrition is common in CRC patients, which results from a combination of malignant disease progression, host tumor responses, anticancer therapies and the direct effects of intestinal obstruction and malabsorption^8–11^. In the past, weight loss and body mass index (BMI) were often used as indicators of poor nutrition and prognosis. However, with improvement in living standards, the numbers of obese patients have increased, pointing to limitations in weight loss and BMI indices as nutritional risk and prognosis indicators. The L3 skeletal muscle index (L3SMI), measured by Computed Tomography (CT), is a criterion for diagnosing sarcopenia but it is controversial. At present, the diagnostic criteria for sarcopenia in Europe and America are widely used, but there are significant racial specificities and differences in terms of skeletal muscle content in Asian, European and American populations^12^. Therefore, the diagnostic criteria for sarcopenia in Europe and America is not suitable for Asian populations.

This study retrospectively analyzed CRC patients from a south China population in Guangxi, to establish the criteria for L3SMI diagnosis of sarcopenia. We also investigated the value of L3SMI in evaluating preoperative nutrition risk status and long-term prognosis of CRC patients. These approaches might provide scientific basis for nutritional intervention and prognostic guidance for CRC patients.

## Materials and Methods

### Study population

CRC patients who experienced surgery in the department of Colorectal and Anal Surgery, the First Affiliated Hospital of Guangxi Medical University from January 2012 to December 2014, were retrospectively analyzed. The inclusion criteria were: (1) The pathological diagnosis of the primary lesion was CRC; (2) The patient had undergone radical resection; (3) A whole abdominal CT scan or enhanced examination was performed within 30 days before surgery and (4) Complete clinical and follow-up data. The exclusion criteria were: (1) Patients who died during the perioperative period; (2) patients with uncertain primary tumor sites; (3) patients with preoperative metastasis and undergoing palliative surgery and (4) missing clinicopathological data and patients lost to follow-up.

This retrospective study was approved by the Hospital Ethics Committee of the First Affiliated Hospital of Guangxi Medical University, Guangxi, China and performed in accordance with the principles of the Declaration of Helsinki. The Ethical approval number was 2019(KY-E-101). According to national laws and institutional requirements, the study did not require written informed consent from the participants.

### Data collection

Collected clinicopathological data included gender, age, BMI, prognostic nutritional index (PNI), Nutrition Risk Screening 2002 (NRS2002), tumor-node-metastasis stage (TNM stage), pathology tumor stage (pT stage), pathology node stage (pN stage), tumor location, perineural/vascular invasion, differentiation, pathological type, serum albumin (ALB) levels, preoperative serum CEA levels, and postoperative chemoradiotherapy.

### CT scan image analysis method of L3SMI

**Figure 1:**
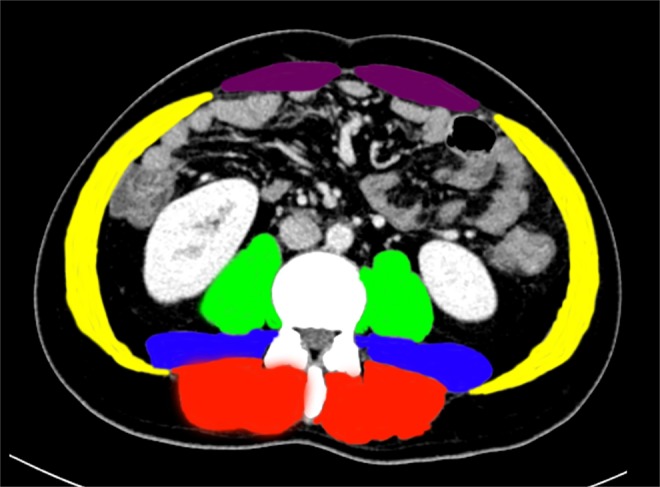
CT scan image analysis method of L3SMIl. Notes: Green is the psoas major, blue is the quadratus lumborum, red is the erector spinae, gold is the transversus abdominis muscle, internal and external oblique muscle, and purple is the rectus abdominis. Abbreviations: L3SMI, L3 skeletal muscle mass index.

CT images were utilizing the 64-slice Siemens Somatom Definition Flash CT instrument of the Light Speed VCT series of the picture archiving and communication system (PACS) before surgery. Plane imaging of the middle and lower third lumbar spine (L3) was selected, and the skeletal muscles were kept separate from other tissues by the Hounsfield unit threshold range of -29 to 150^13,14^. The L3 skeletal muscles included the psoas muscle, the lumbar muscle, the erector spinae, the transversus abdominis muscle, the internal and external oblique muscles, and the rectus abdominis. Two consecutive images were recorded in the L3 plane. The sum of the cross-sectional areas of all skeletal muscles was calculated, averaged and divided by the square of the height. The formula used was: L3SMI = L3 skeletal muscle cross-sectional area (cm^2^)/height^2^(m^2^) (Fig. 1).

### Definition and diagnostic criteria for sarcopenia

Previous studies by Prado *et al*.^15^ and Vledder *et al*.^16^ have proposed the L3SMI criteria for the diagnosis of sarcopenia, which is generally acknowledged. However, as we all know, Westerners usually have a larger physique and a higher BMI than Eastern, which may make these standards unsuitable for the Eastern population. In this study, we divided CRC patients into four groups (Q1, Q2, Q3, Q4) by referring to the L3SMI gender quartile method of Miyamoto *et al*.^7^.

### Nutritional risk grouping

Nutritional risk screening 2002 (NRS 2002) was proposed by Kondrup *et al*.^17^ to assess the extent of malnutrition in patients. It is currently applied to the nutritional risk assessment of patients with various chronic diseases, including various malignancies^18,19^. In our study, CRC patients were divided into a nutritional risk group and non-nutritional risk group according to NRS 2002. The NRS 2002 covers three aspects: (1) disease severity score: range 0 to 3 points, the lowest score for patients with gastrointestinal cancer was 1 point; (2) nutritional status damage degree: range 0 to 3 points, mainly based on BMI, recent diet and weight changes; (3) age factor: age ≥ 70 years is 1 point. The total score is the sum of the three scores, and ≥ 3 points suggests a nutritional risk.

### Follow-up

Patients were primarily followed up by telephone and outpatient clinic visits, patients were reexamined every 3 months within 2 years after surgery and then every 3 to 6 months thereafter. The follow-up included blood tests, checking tumor biomarkers, image diagnosis (X-rays, positron emission tomography, computed tomography) and colonoscopy. The final follow-up visit occurred on August 1, 2019. Loss of follow-up was defined as the absence of outpatient clinic visits or the failure of telephone contact for more than 6 months. Disease-free survival (DFS) was defined as the interval from cancer resection to recurrence, metastasis, death or the last follow-up. The overall survival (OS) was defined as the interval from cancer resection to death or the last follow-up.

### Statistical analysis

Correlations between L3SMI and clinicopathological data were performed using the chi-square test or analysis of variance. The Logistic regression was used to assess the associations between L3SMI and nutritional risk. Survival curves were estimated by the Kaplan-Meier method and log-rank tests were used to compare differences between the L3SMI groups. The Cox proportional hazard model was used for univariate analysis, multivariate analysis and subgroup multivariate analysis to assess relationships between L3SMI and prognosis. The nomogram for predicting nutritional risk was established using the Logistic regression model. The nomograms for predicting 1-5 years DFS and 1-5 years OS were established by the Cox proportional hazards model. The p < 0.05 (two-sided) was considered statistically significant. All data were analyzed using IBM SPSS 24.0 (IBM Corp, Armonk, NY, USA) and R Version 3.5.3(https://www.r-project.org/).

## Results

### Patients

A total of 500 patients with TNM I-III stages were collected, of which 41 patients were unable to collect preoperative CT images, 20 patients did not have complete clinicopathological data, and 39 patients lost contact during follow-up. Finally, 400 patients were enrolled in the study. The median follow-up time was 63 months (range 6–80 months). CRC patients were split into four groups using different gender quartile methods of L3SMI: Q1 (male: 21.04 cm^2^/m^2^–38.89 cm^2^/m^2^, n = 64; female: 21.96 cm^2^/m^2^–33.28 cm^2^/m^2^, n = 35), Q2 (male: 39.04 cm^2^/m^2^–45.26 cm^2^/m^2^, n = 64; female: 33.58 cm^2^/m^2^–37.14 cm^2^/m^2^, n = 35), Q3 (male: 45.42 cm^2^/m^2^–50.35 cm^2^/m^2^, n = 64; female: 37.37 cm^2^/m^2^–40.74 cm^2^/m^2^, n = 35) and Q4 (male: 50.69 cm^2^/m^2^–66.69 cm^2^/m^2^, n = 65; female: 40.91 cm^2^/m^2^–57.25 cm^2^/m^2^, n = 38). The outcome events of overall survival were 136 patients, while the outcome events of disease-free survival were 142 patients.

### The correlation between quartiles L3SMI and factors

The chi-square test or analysis of variance was used to detect correlations between L3SMI and clinicopathological factors, including gender, age, ALB, BMI, PNI, NRS2002, pT stage, pN stage, perineural/vascular invasion, tumor location, differentiation, pathological type, and CEA levels. From our analyses, L3SMI was associated with age (p < 0.001), ALB (p < 0.001), BMI (p < 0.001), PNI (p < 0.001) and NRS2002 (p < 0.001) (Table 1).

**Table 1:** Correlation analysis between clinicopathological indicators of colorectal cancer and four groups of L3SMI.

Features	Case (%)	L3SMI (quartile)(42.77 ± 8.61)				*Χ*^*2*^*/F*	*P*
		Q1(33.50 ± 3.87)	Q2(39.79 ± 3.64)	Q3(44.72 ± 4.44)	Q4(52.65 ± 7.03)		
All patients	400	99	99	99	103		
Gender(Male/Female)	257/143	64/35	64/35	64/35	65/38	0.079	0.994
Age (Years)	57.80 ± 13.09	63.36 ± 13.91	59.38 ± 13.13	56.47 ± 11.20	52.21 ± 11.51	14.340	<0.001
ALB(g/L)	37.01 ± 4.38	35.13 ± 3.97	36.85 ± 4.21	37.77 ± 4.61	38.22 ± 4.13	10.410	<0.001
BMI(Low/Normal/High)	55/232/113	36/59/4	15/69/15	2/63/34	2/41/60	126.784	<0.001
PNI(<45/≥45)	161/239	54/45	45/54	34/65	28/75	18.275	<0.001
NRS2002(Nutrition risk/Non-nutrition risk)	86/314	54/45	24/75	3/96	5/98	101.415	<0.001
pT stage(T1-2/T3-4)	118/282	33/66	23/76	27/72	35/68	3.800	0.284
pN stage(N0/N1/N2)	230/115/55	59/29/11	58/26/15	57/23/19	56/37/10	7.385	0.287
pTNM stage(I/II/III)	88/142/170	24/34/41	19/40/40	21/36/42	24/32/47	2.355	0.884
Tumor location(Rectal/Colon)	212/188	47/52	48/51	60/39	57/46	4.549	0.208
Perineural/vascular invasion(Negative/Positive)	333/67	83/16	81/18	80/19	89/14	1.330	0.722
Pathological type(Protrude/Infiltrating/Ulcerative)	77/44/279	25/11/63	17/11/71	18/10/71	17/12/74	3.357	0.763
Differentiation(Poor/Medium and High)	43/357	8/91	16/83	12/87	7/96	5.629	0.131
CEA(<5 ng/ml/≥5 ng/ml)	265/135	68/31	58/41	51/38	78/25	9.936	0.019

Table Note: body mass index (BMI) = body weight (kg)/height (m) square.According to China’s BMI standard, BMI < 18.5 is low, 18.5 ≤ BMI < 24 is normal, ≥ 24 is high; prognostic nutritional index(PNI) = albumin level (g/L) + 5 × lymphocyte count (109/L). According to previous studies, PNI < 45 was considered to be low, and PNI ≥ 45 was considered normal.

### The modified categories of L3SMI

**Figure 2:**
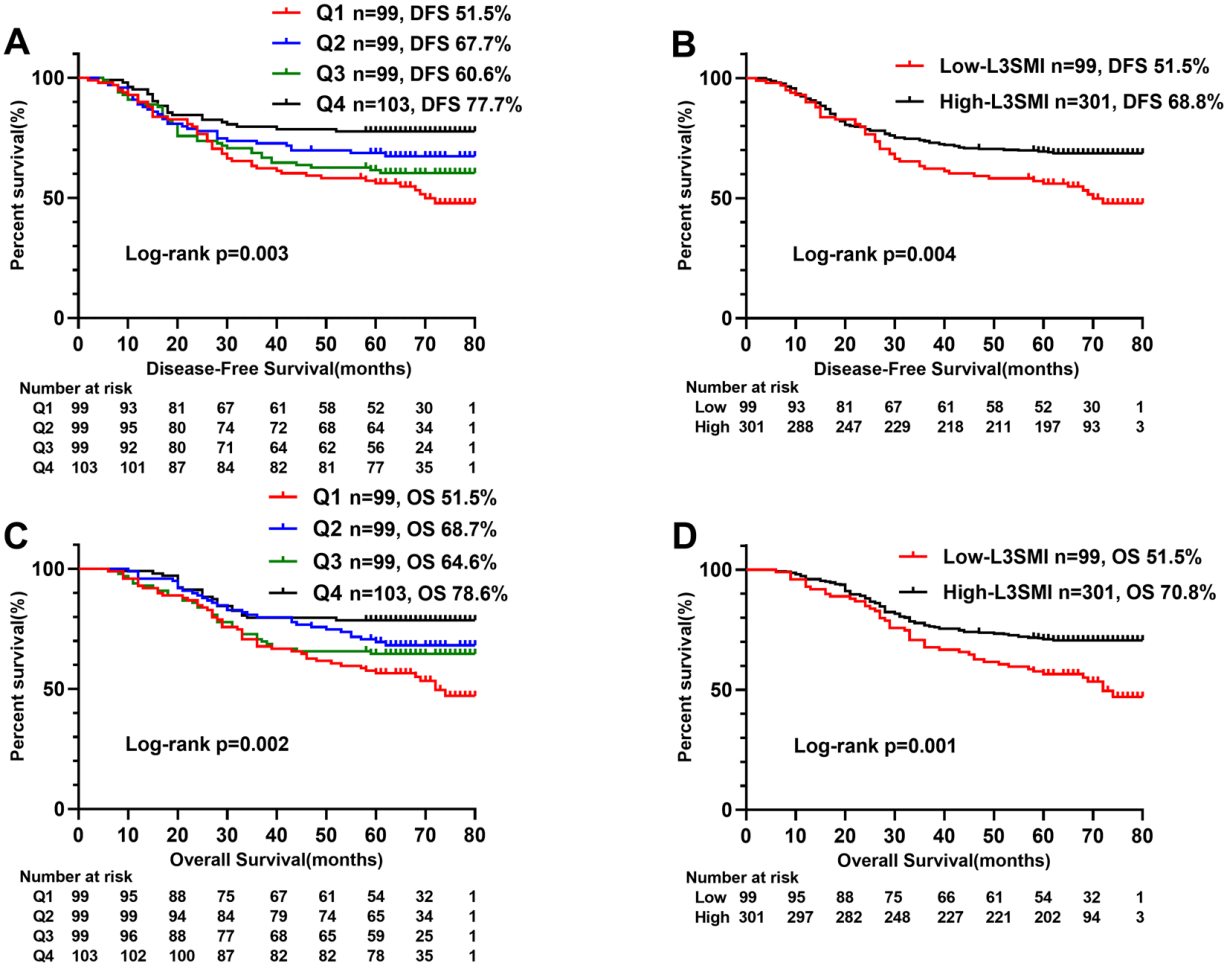
Kaplan-Meier survival curve of the gender quartile method (Q1-4) of L3SMI in CRC patients. Notes: A, disease-free survival curve of the quartile (Q1-4) of L3SMI; B, disease-free survival curve of high- and low-L3SMI; C, overall survival curve of the quartile (Q1-4) of L3SMI; D, overall survival curve of high- and low-L3SMI. Abbreviations: L3SMI, L3 skeletal muscle mass index; Q1, represents the group of sarcopenia; Q2-4, represent the group non-sarcopenia.

We divided L3SMI into four groups: Q1, Q2, Q3 and Q4 by gender quartile methods. We performed a Cox regression analysis on L3SMI (Q1–Q4). In the univariate and multivariate analysis, compared with Q1 patients, the disease-free survival rate of Q2 and Q4 patients were significantly higher (respectively, p < 0.05), while the disease-free survival rate of Q3 patients was not significantly improved (p > 0.05). In univariate and multivariate analysis, compared with Q1 patients, the overall survival rate of Q4 and Q2 patients were significantly higher (respectively, p < 0.05), while the overall survival rate of Q3 patients was not significantly improved (p > 0.05). Coupled with the survival curve in Fig. 2, we found that Q1 was more differentiated from other groups, and thus inferred that Q1 group was more suitable as a threshold for sarcopenia. Based on these analyses, we established the dichotomous L3SMI variable, combining Q2, Q3 and Q4 groups as the high-L3SMI group, and placing the lowest quarter (Q1) group into the low-L3SMI group (Table 2).

**Table 2:** Comparison of classification methods of L3SMI.

L3SMI	Disease-free survival				Overall survival			
	Univariate		Multivariate		Univariate		Multivariate	
	HR (95%CI)	p	HR (95%CI)	p	HR (95%CI)	p	HR (95%CI)	p
Q1	1	0.004	1	0.001	1	0.003	1	<0.001
Q2	0.637(0.407–0.996)	0.048	0.510(0.321–0.811)	0.004	0.785(0.366–1.682)	0.026	0.471(0.295–0.752)	0.002
Q3	0.799(0.523–1.219)	0.297	0.688(0.449–1.053)	0.085	0.789(0.373–1.667)	0.147	0.642(0.414–0.996)	0.048
Q4	0.411(0.250–0.676)	<0.001	0.392(0.237–0.647)	<0.001	0.474(0.231–0.976)	<0.001	0.376(0.226–0.625)	<0.001
Low L3SMI (Q1)	1	0.005	1	<0.001	1	0.001	1	<0.001
High L3SMI (Q2-4)	0.606(0.428–0.859)		0.528(0.371–0.752)		0.566(0.398–0.804)		0.493(0.345–0.704)	

### Factors affecting preoperative nutritional risk

CRC patients were divided into a nutritional risk group and a non-nutrition risk group, according to NRS2002. Logistic regression analysis was then performed to analyze factors related to nutrition risk. Univariate logistic regression analysis indicated that age, ALB, BMI, PNI, pathological type and L3SMI were associated with nutrition status. The above factors were included in multivariate Logistic regression analysis, and only PNI (p = 0.013), BMI (p < 0.001), L3SMI (p < 0.001) were observed as independent factors affecting nutritional risk in CRC patients (Table 3).

**Table 3:** Univariate and multivariate logistic regression analysis of influencing factors of nutritional risk in CRC patients.

Feature	Univariate analysis		Multivariate analysis	
	HR (95%CI)	*p*	HR (95%CI)	*p*
Gender (Female)	1.230(0.753–2.009)	0.409		
Age (≥60)	1.742(1.074–2.828)	0.025	1.742(1.074–2.828)	0.237
ALB(≧40 g/L)	0.102(0.031–0.332)	<0.001	0.318(0.051–1.965)	0.218
BMI		<0.001		<0.001
Low	1.000		1.000	
Normal	0.002(0.000–0.018)		0.002(0.000–0.016)	
High	0.001(0.000–0.009)		0.002(0.000–0.017)	
PNI(≥45)	0.252(0.152–0.418)	<0.001	0.338(0.146–0.782)	0.011
L3SMI(Low L3SMI)	10.088(5.883–17.298)	<0.001	6.214(2.632–14.673)	<0.001
T stage(T3-4)	0.777(0.467–1.295)	0.333		
N stage		0.994		
N0	1.000			
N1	1.026(0.596–1.768)			
N2	1.026(0.505–2.104)			
pTNM stage		0.560		
I stage	1.000			
II stage	0.704(0.372–1.335)			
III stage	0.835(0.456–1.528)			
Tumor location(Colon)	1.393(0.863–2.248)	0.175		
Perineural/vascular invasion(Positive)	1.301(0.706–2.396)	0.399		
Pathological type		0.283		
Protrude type	1.000			
Infiltrating type	1.069(0.463–2.467)			
Ulcerative type	0.684(0.379–1.233)			
Differentiation (Medium/High)	1.223(0.545–2.744)	0.625		
CEA(>5 ng/ml)	0.678(0.389–1.180)	0.170		

### The value of L3SMI in Long-term outcomes

**Figure 3:**
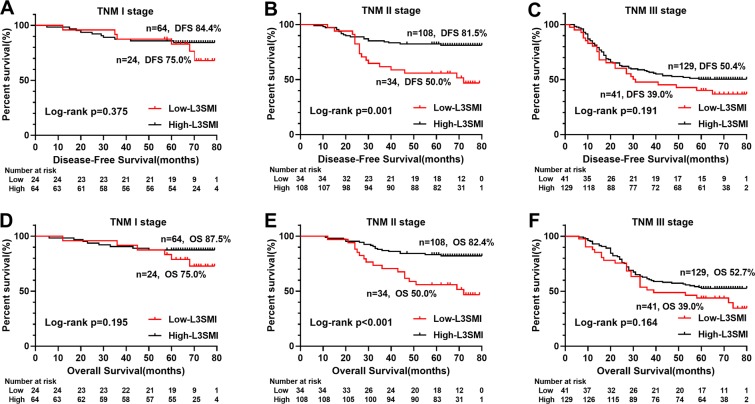
Stratified Kaplan-Meier survival curves of two groups of L3SMI based on different TNM stages. Notes: A, disease-free survival of L3SMI in TNM I; B, disease-free survival of L3SMI in TNM II; C, disease-free survival of L3SMI in TNM III; D, overall survival of L3SMI in TNM I; E, overall survival of L3SMI in TNM II; F, overall survival of L3SMI in TNM III. Abbreviations: L3SMI, L3 skeletal muscle mass index.

**Figure 4:**
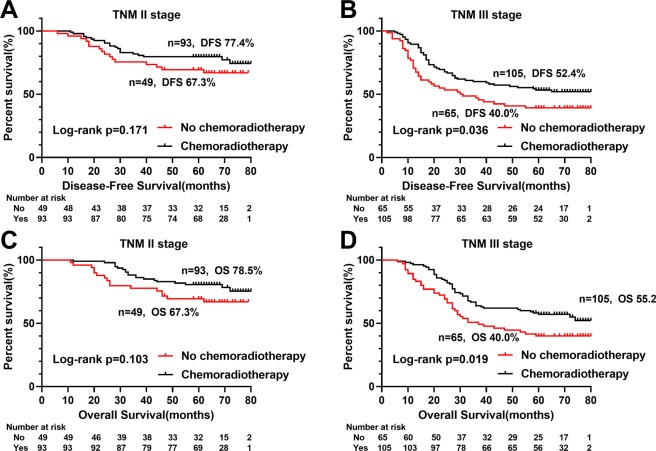
Stratified Kaplan-Meier survival curves of postoperative chemoradiotherapy based on different TNM stages. Notes: A, disease-free survival of postoperative chemoradiotherapy in TNM II; B, disease-free survival of postoperative chemoradiotherapy in TNM III; C, overall survival of postoperative chemoradiotherapy in TNM II; D, overall survival of postoperative chemoradiotherapy in TNM III; patients with TNM I stage received few chemoradiotherapy and were not shown. Abbreviations: L3SMI, L3 skeletal muscle mass index.

Kaplan-Meier analysis showed significant differences in survival curves among the quartiles L3SMI groups (respectively, DFS (p = 0.003) and OS (p = 0.002)) (Fig. 2A,C), especially DFS (p = 0.005) and OS (p = 0.001) of low-L3SMI significantly lower than the high-L3SMI (Fig. 2B,D). Further stratified analysis of TNM stages showed statistically significant differences between low-L3SMI and high-L3SMI for TNM II stage CRC patients in DFS (p < 0.001) and OS (p < 0.001), respectively (Fig. 3). In addition, it is well known that postoperative chemoradiotherapy is helpful to improve the prognosis of patients. In this study, we performed a survival analysis of postoperative chemoradiotherapy based on TNM stage stratification, and found that postoperative chemoradiotherapy was beneficial to postoperative CRC patients with TNM III stage both in DFS (p = 0.036) and OS (p = 0.019) (Fig. 4).

**Figure 5:**
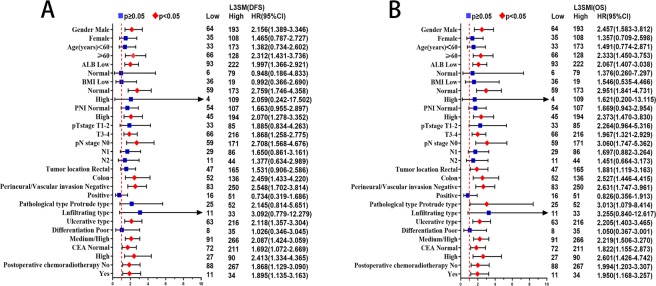
Subgroup survival analysis in colorectal cancer patients. Notes: A, subgroup disease-free survival analysis of L3SMI; B, subgroup overall survival analysis of L3SMI. Abbreviations: L3SMI, L3 skeletal muscle mass index; DFS, disease-free survival; OS, overall survival.

In univariate survival analysis, L3SMI, pT stage, pN stage, differentiation and CEA levels were associated with DFS and OS. In multivariate survival analysis, L3SMI (p < 0.001), pT stage (p = 0.032), pN stage (p < 0.001) and CEA levels (p = 0.047) were independent prognostic factors for DFS, while L3SMI (p < 0.001), pT stage (p = 0.035), pN stage (p < 0.001) were independent prognostic factors for OS (Table 4). In multivariate subgroup survival analysis of DFS, low L3SMI was an independent risk factor for prognosis in males, ≧60 years, low ALB, normal BMI, high PNI, T3–4 stage, pN0 stage, colon cancer, negative perineural/vascular invasion, medium/high differentiation, ulcerative type, normal CEA and high CEA levels, postoperative chemoradiotherapy and no postoperative chemoradiotherapy (Fig. 5A). In multivariate subgroup survival analysis of OS, low L3SMI was an independent risk factor for prognosis in male, ≥60 years, low ALB, normal BMI, high PNI, T3–4 stage, pN0 stage, rectal and colon cancer, negative perineural/vascular invasion, protrude type and ulcerative type, medium/high differentiation, normal and high CEA levels, postoperative chemoradiotherapy and no postoperative chemoradiotherapy (Fig. 5B).

**Table 4:** Univariate and multivariate survival analysis of clinicopathological characteristics in CRC patients.

Feature	Disease-free survival				Overall survival			
	Univariate		Multivariate		Univariate		Multivariate	
	HR (95%CI)	*p*	HR (95%CI)	*p*	HR(95%CI)	*p*	HR(95%CI)	*p*
Gender (Female)	0.876(0.617–1.242)	0.457			0.866(0.605–1.241)	0.434		
Age (≥60)	1.270(0.913–1.768)	0.155			1.416(0.935–1.988)	0.094		
ALB (≧40 g/L)	0.944(0.624–1.428)	0.785			0.882(0.575–1.352)	0.564		
BMI		0.600				0.738		
Low	1.000				1.000			
Normal	1.064(0.654–1.730)				1.023(0.627–1.667)			
High	0.869(0.502–1.505)				0.873(0.503–1.517)			
PNI (≥45)	1.001(0.716–1.399)	0.995			0.997(0.708–1.405)	0.987		
NRS2002 (Non-nutrition risk group)	1.120(0.762–1.647)	0.565			1.140(0.770–1.687)	0.513		
L3SMI (Low L3SMI)	1.649(1.164–2.335)	0.005	1.894(1.330–2.698)	<0.001	1.768(1.244–2.514)	0.001	2.030(1.420–2.902)	<0.001
pT stage (T3-4)	2.312(1.501–3.561)	<0.001	1.638(1.043–2.572)	0.032	2.351(1.501–3.681)	<0.001	1.657(1.036–2.648)	0.035
pN stage		<0.001		<0.001		<0.001		<0.001
N0	1.000		1.000		1.000		1.000	
N1	2.096(1.415–3.105)		1.739(1.153–2.623)		2.049(1.370–3.066)		1.707(1.120–2.602)	
N2	6.034(4.012–9.074)		4.530(2.855–7.187)		5.923(3.904–8.985)		4.486(2.800–7.188)	
Tumor location (Colon)	0.877(0.630–1.222)	0.438			0.813(0.579–1.142)	0.233		
Perineural/vascular invasion (Positive)	2.125(1.460–3.093)	<0.001	1.319(0.887–1.960)	0.171	2.002(1.362–2.942)	<0.001	1.247(0.831–1.871)	0.286
Pathological type		0.477				0.539		
Protrude type	1.000				1.000			
Infiltrating type	1.300(0.687–2.462)				1.349(0.708–2.568)			
Ulcerative type	1.322(0.841–2.077)				1.281(0.807–2.035)			
Differentiation(Medium/High)	0.453(0.292–0.703)	<0.001	0.744(0.462–1.198)	0.224	0.443(0.285–0.689)	<0.001	0.729(0.450–1.181)	0.200
CEA(≥5 ng/ml)	1.666(1.195–2.324)	0.003	1.425(1.005–2.020)	0.047	1.563(1.111–2.198)	0.010	1.387(0.970–1.983)	0.073
Postoperative chemoradiotherapy(Yes)	0.920(0.661–1.279)	0.618			0.879(0.628–1.230)	0.451

### The nomogram

**Figure 6:**
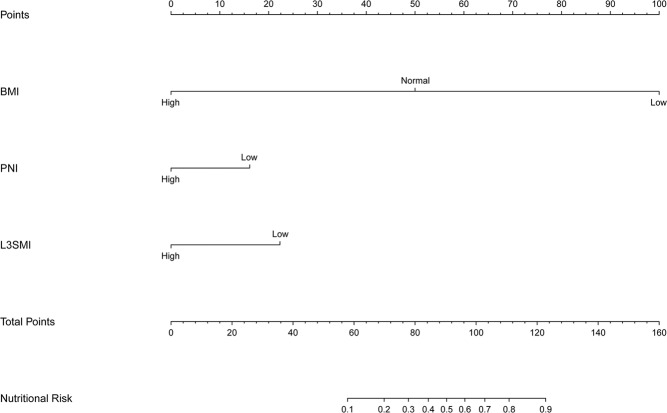
The nutritional risk nomogram. Abbreviations: BMI, body mass index; PNI, prognostic nutritional index; L3SMI, L3 skeletal muscle mass index.

**Figure 7:**
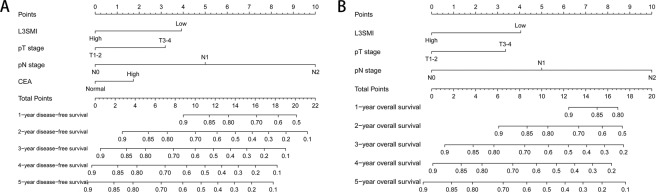
The survival risk nomograms. Notes: A, the survival risk model of DFS; B, the survival risk model of OS. Abbreviations: BMI, body mass index; PNI, prognostic nutritional index; L3SMI, L3 skeletal muscle mass index; DFS, disease-free survival; OS, overall survival.

A nomogram was established to assess the preoperative nutrition risk of CRC patients, after adjustment by Logistic regression model. BMI, PNI and L3SMI were considered into the nutritional risk model (Fig. 6). The nutritional risk can be predicted individually for CRC patient by calculating scores for each factor. Two nomograms were established to assess the survival risk of CRC patients. After adjustment by Cox proportional hazards model, pT stage, pN stage, L3SMI and CEA levels were considered into the survival risk model of DFS, and pT stage, pN stage and L3SMI were considered into the prognostic risk model of OS. The DFS (Fig. 7A) and OS (Fig. 7B) of 1–5 years can be predicted individually for CRC patient by calculating scores for each factor.

## Discussion

In recent years, many studies have shown that systemic inflammatory responses and nutritional status are closely associated with the prognosis of many malignancies^20,21^. Systemic inflammation involves cancer cell secretion of inflammatory cytokines, which reflects the biological activities of tumor cells. Sarcopenia is reported to be closely related to systemic inflammation^22^, which is a syndrome that increases the risk of adverse events and is characterized by decreased skeletal muscle mass and decreased muscle strength or function^8^. The development of sarcopenia may be the result of combination of muscle disuse, systemic inflammation, malnutrition and dyscrasia^23,24^. Skeletal muscle generates autocrine, paracrine and endocrine effects, secreting cytokines (IL-6, IL-8, IL-15) and other inflammatory markers including CRP, and TNF-alpha which lead to systemic inflammatory effects^25^. Rong *et al*., observed that inflammatory factor Il-6 promoted the catabolism of skeletal muscle, while TNF-alpha directly decreased muscle proteins, resulting in muscle loss and atrophy^26^.

The L3SMI measured by computed tomography (CT) is an extensive and accurate method for assessing skeletal muscle mass. CT provides important quantitative information on muscle composition and distribution^27^, through which high quality images, spatial accuracy and positional features enable high-precision measurement of fat and muscle content^28^. However, currently there are no any uniform standards for the diagnosis of sarcopenia. Based on the survival rates of obese cancer patients in European populations, diagnostic criteria for sarcopenia was proposed in 2008 (females, SMI < 38.5 cm^2^/m^2^; males, SMI < 52.4 cm^2^/m^2^)^15^. Thereafter, diagnostic criteria based on BMI and gender differences in 1473 tumor patients was recorded in 2013 (males, BMI ≥ 25 kg/m^2^, SMI < 53 cm^2^/m^2^; males, BMI < 25 kg/m^2^, SMI < 43 cm^2^/m^2^; females, regardless of BMI, SMI < 41 cm^2^/m^2^)^29^. However, some studies have reported that as for the comparison of muscle mass, the Asian population is significantly lower than the Western population about 17%^12^. Because these standards were based on Western populations, they were not applicable to Asian populations. Recently, a study investigating 937 Chinese gastric cancer patients suggested the ideal cut-off value for Chinese should be; females ≤ 34.9 cm^2^/m^2^ and males ≤ 40.8cm^2^/m^2^, however these metrics were based on gastric cancer criteria, and not CRC^30^. Many studies also classify muscle reduction by SMI median, tertile or quartile methods^31–36^, In one study, 220 CRC patients were split into groups (Q1, Q2, Q3, Q4) by the sex quartile method, with the lowest group identified as the sarcopenia group^7^. This method reduced the gender difference and interference effects from different races. The classification method was adopted in our study. According to the gender quartile method, we generated the following values for the diagnosis of sarcopenia in Chinese CRC population: males L3SMI ≤ 38.89 cm^2^/m^2^, females L3SMI ≤ 33.28 cm^2^/m^2^.

Currently, the most commonly nutrition risk screening tool is NRS2002, which is approved by the Chinese Medical Association and the European Society of Parenteral Enteral Nutrition (ESPEN)^37^. NRS2002 was used to evaluate nutrition risk in our study. The multivariate logistic regression analyses showed that low L3SMI was an independent risk factor for malnutrition in preoperative CRC patients. Some researches have confirmed that decreased L3SMI, inadequate energy intake and inadequate energy-use can lead to increasing risk of malnutrition-related disease^38^. With the increasing number of obese patients, the BIM as a nutrition risk and prognostic indicator has obvious shortcomings. In our study, about 18.26% patients with normal and high BMI had sarcopenia, while about 14.33% patients with normal nutrition of NRS2002 had sarcopenia. These patients with malnutrition were usually neglected and were unable to receive standard nutritional interventions, thus leading to poor prognosis. Therefore, L3SMI can be combined with NRS2002 to provide a more comprehensive assessment of the nutritional status of CRC patients during perioperative risk assessment.

Numerous studies have shown that low L3SMI affects the prognosis of Many malignancies^20,23,29,39,40^. In our study, multivariate survival analysis of DFS and OS both suggested that low L3SMI was an independent risk factor for CRC patients. In our further stratified analysis, L3SMI could distinguish the poor prognosis of patients with TNM stage II, while there was a tendency to distinguish the poor prognosis in patients with TNM III stage, but it was not statistically significant. Therefore, we speculated that L3SMI might be more suitable for prognostic evaluation of CRC patients with TNM II stage. In addition, we found that postoperative chemoradiotherapy for CRC patients with TNM III stage had a survival benefit. Based on these findings, we believed that it might be the benefit of postoperative chemoradiotherapy for TNM stage III patients that weakens the impact of L3SMI on the prognosis. Therefore, improving sarcopenia and enhancing nutrition may prolong survival in patients with TNM stage II, while improving sarcopenia and increasing postoperative adjuvant chemotherapy may benefit patients with TNM stage III.

We also established three nomograms. In the nutritional risk model, we found that the contributions of BMI, PNI and L3SMI were decreased with unfavorable disease stages. By applying this model, we could forecast nutritional risk of each CRC patients. Patients with lower points had a much better nutritional status than those with higher points. In the DFS survival risk model, we found increased contributions of pT stage, pN stage, L3SMI and CEA levels, with advancing disease stage. In the OS survival risk model, we found increased contributions of pT stage, pN stage, and L3SMI with advancing disease stage. By applying those models, we could forecast the 1-5 years survival of each CRC patients. Patients with lower points had much better survival rates, than those with higher points.

This study suggested that the improvement of sarcopenia might be an effective way to improve the prognosis of CRC patients. Preoperative resistance training, adequate nutrition intake can be used to maintain muscle mass. Patients with high nomograms scores could be encouraged to enhance postoperative nutritional support and early out-of-bed activity to improve long-term outcomes. In terms of study limitations. First, we only defined sarcopenia by muscle mass, ignoring changes in muscle function. It is necessary to increase the assessment of muscle function in future prospective studies. Second, this retrospective study was based at a single-institution with limited data, so the findings must be verified in multiple-centers or larger cohort studies in the future. Finally, the nomograms were constructed based on a limited sample of patients, therefore these must be validated in larger, more widespread populations. However, this study had sufficient samples, and we screened patients strictly according to the inclusion and exclusion criteria, and developed a strict follow-up strategy to ensure that each patient is followed up to 5 years, which guaranteed the validity of survival data. In addition, most of the CT images of patients were collected within 1 week before operation, which to some extent ensured that the L3SMI of the patients would not deviate due to the long interval between the collection and the operation, thus ensuring the validity of the measurement results. What’s more, we determined the cut-off value of sarcopenia by gender quartile method, which was similar to the cut-off value of other tumors in the Chinese population^30^, indicating that this method has good practicability and can provide certain reference for the follow-up studies on sarcopenia in the Chinese population. Therefore, we considered that the results of this study are reproducible and reliable.

In conclusion, this retrospective study firstly revealed that the CT diagnostic criteria of sarcopenia in South China CRC population was male ≤38.89cm^2^/m^2^, female ≤33.28cm^2^/m^2^. L3SMI is a useful supplement for screening preoperative nutritional risk and diagnosing sarcopenia, and a potential clinical indicator that can be used to predict the prognosis of CRC patients, especially TNM stage II patients. Nomograms based on L3SMI can be used to screen patients with high nutritional risk and poor prognosis, providing scientific reference for early individualized nutritional intervention, postoperative follow-up and treatment stratification.

## Data Availability

The data-sets used and/or analyzed during the current study are available from the corresponding author on reasonable request.
